# A case of papillary renal cell carcinoma with para‐aortic lymph node metastasis that was completely resectable after treatment with combination therapy comprising lenvatinib and pembrolizumab and robot‐assisted radical nephrectomy

**DOI:** 10.1002/iju5.12830

**Published:** 2025-01-29

**Authors:** Takayuki Hosoi, Shunsuke Yoshioka, Akari Hiraguri, Yusuke Kirihana, Tomoyuki Koguchi, Makoto Tamaki, Chiharu Irisawa

**Affiliations:** ^1^ Urology Takeda General Hospital Aiduwakamatu Fukushima Japan; ^2^ Urology Ohta Nishinouchi Hospital Koriyama Fukushima Japan; ^3^ Urology Aizu Clinic Aiduwakamatu Fukushima Japan; ^4^ Urology Irisawa Clinic Aiduwakamatu Fukushima Japan

**Keywords:** cytoreductive nephrectomy, lenvatinib, lymphatic metastasis, papillary renal cell carcinoma, pembrolizumab

## Abstract

**Introduction:**

Combination therapy comprising immune checkpoint inhibitors and molecular targeted therapies can be effective for metastatic non‐clear cell renal cell carcinoma. We describe a patient with metastatic papillary renal cell carcinoma that was completely pathologically resectable after combination treatment comprising lenvatinib and pembrolizumab and robot‐assisted radical nephrectomy.

**Case presentation:**

A 35‐year‐old man was referred to our department for examination of a renal tumor that was diagnosed as left renal cell carcinoma with lymph node metastasis (cT3aN1M0). Because metastasis affected the renal artery, combination therapy comprising lenvatinib and pembrolizumab was initiated. After 6 months of treatment, the patient underwent robot‐assisted radical nephrectomy. The pathological results indicated papillary renal cell carcinoma with no viable cells in the lymph nodes.

**Conclusion:**

Combination therapy comprising lenvatinib and pembrolizumab for unresectable papillary renal cell carcinoma may effectively allow radical resection and deferred cytoreductive nephrectomy.

**Abbreviations & Acronyms**
ccRCC: clear cell renal cell carcinoma
CN: cytoreductive nephrectomy
CT: computerized tomography
FGFR2: fibroblast growth factor receptor‐2
ICI: immune checkpoint inhibitor
nccRCC: non‐clear cell renal cell carcinoma
RCC: renal cell carcinoma
TKI: tyrosine kinase inhibitor

**Keynote message**

Therapy comprising immune checkpoint inhibitors and tyrosine kinase inhibitors is often used to treat metastatic renal cell carcinoma. Combination therapy comprising lenvatinib and pembrolizumab was effective for this case of papillary renal cell carcinoma. Complete resection was achieved after deferred cytoreductive nephrectomy.

## Introduction

Two ICIs in combination (IO‐IO combination therapy) as well as one ICI and one TKI in combination (IO‐TKI therapy) are useful for the treatment of metastatic or unresectable RCC.^1^, ^2^, ^3^, ^4^, ^5^ However, the efficacy of these combination therapies for the treatment of nccRCC has not been sufficiently investigated. Recently, the efficacy and safety of combination therapy comprising lenvatinib and pembrolizumab for nccRCC have been reported.^6^ We report a case of papillary RCC that was completely resected after primary treatment with lenvatinib and pembrolizumab combination therapy and deferred CN. This case had been considered ineligible for radical resection because of lymph node metastasis to the renal hilus.

## Case presentation

**Fig. 1:**
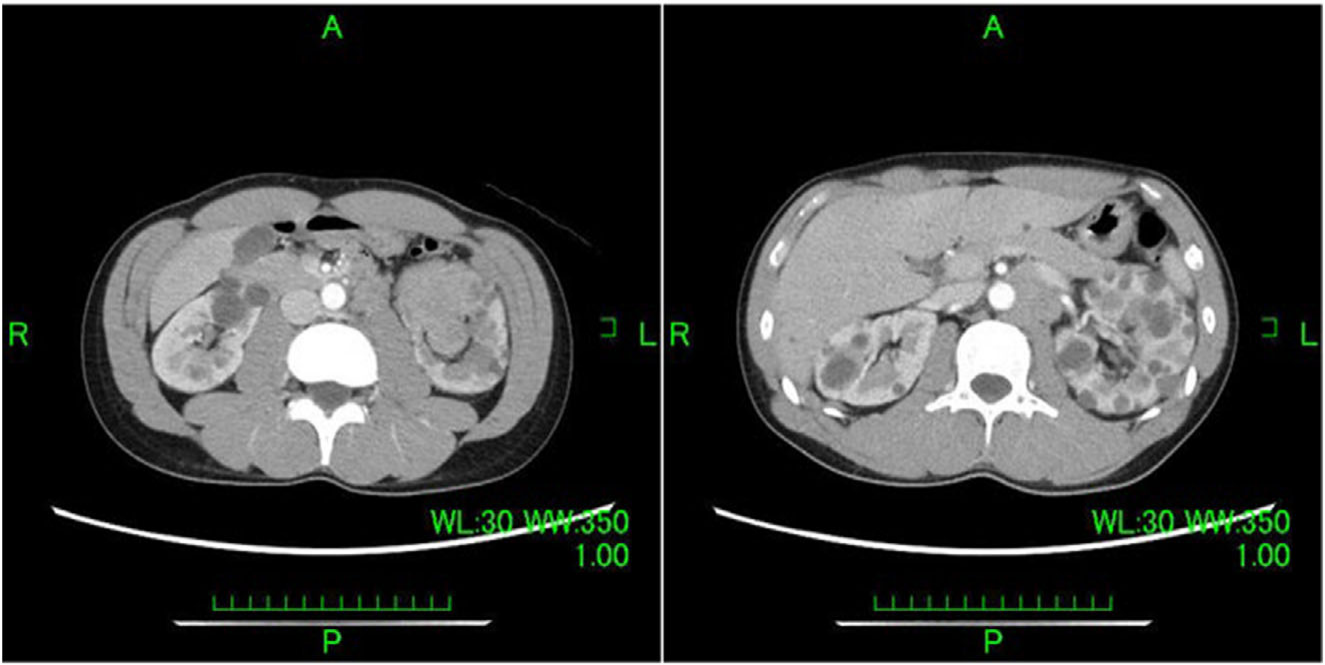
CT image of clear cell renal cell carcinoma showing a progressively increasing pattern of early dark staining and washout. Therefore, papillary renal cell carcinoma and renal pelvic cancer were possible diagnoses. The tumor protrudes from the renal parenchyma to the renal pelvis; therefore, it is considered the cause of gross hematuria. Lymph node metastases with a maximum diameter of 40 mm are mainly observed in the renal portals. The renal artery is buried in the lymph nodes, and the renal vein drains ventrally.

**Fig. 2:**
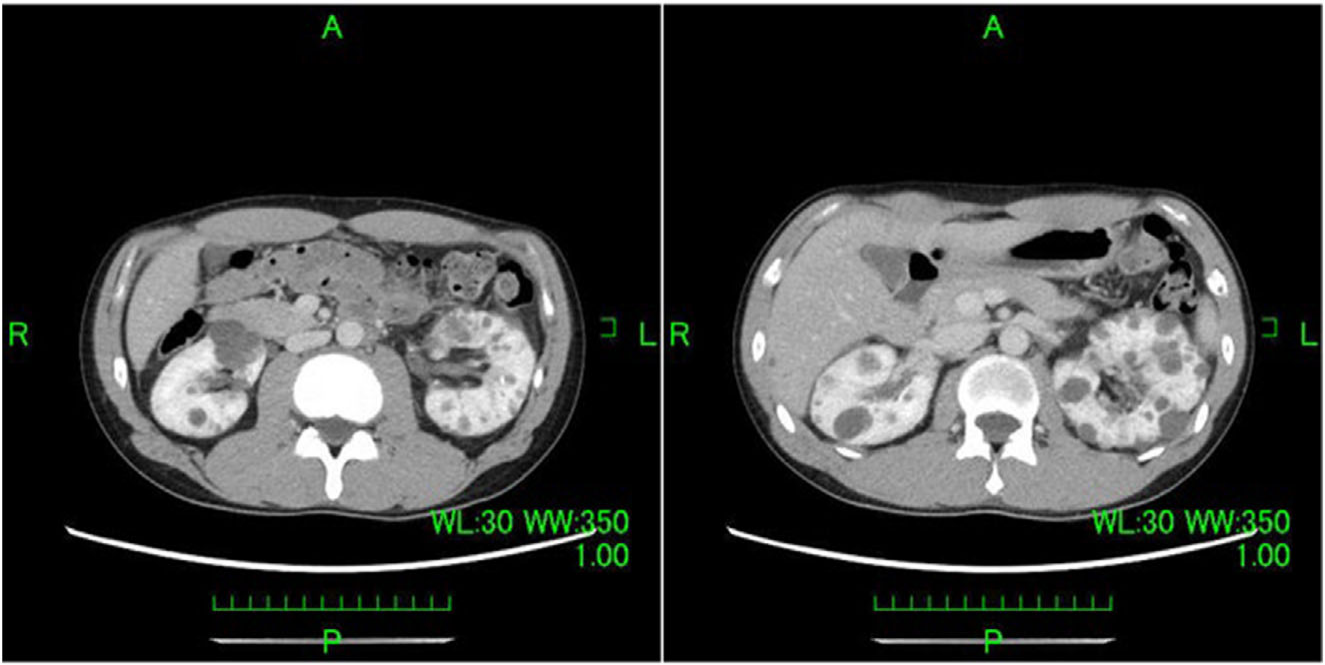
Six months after treatment initiation, a CT evaluation revealed that the mass lesion in the left renal parenchyma that extended to the renal pelvis and para‐aortic lymph nodes was smaller.

**Fig. 3:**
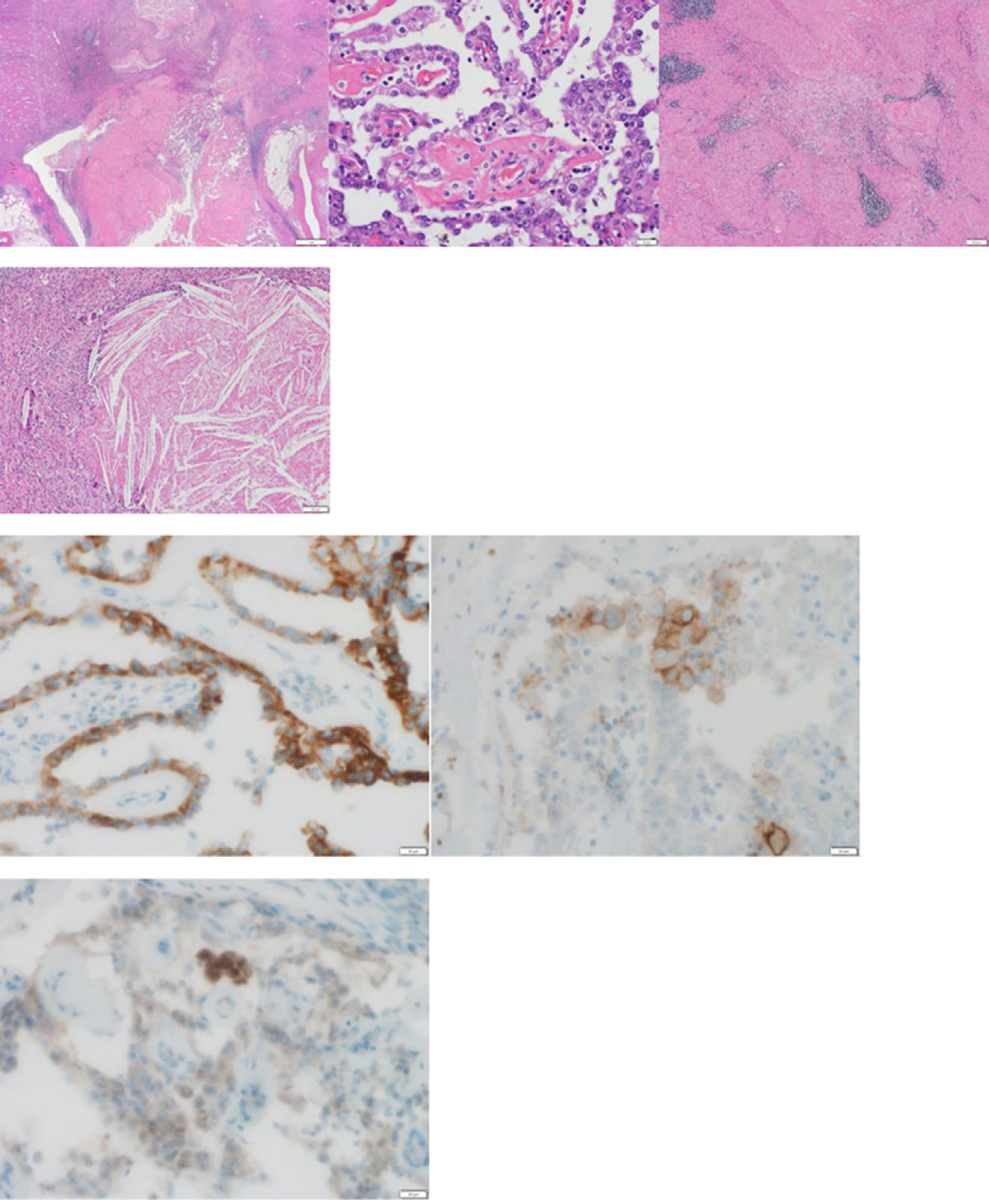
Papillary proliferation of nucleus‐like tumor cells with fibrous vessels. The surrounding area is highly infiltrated with vitreous, macrophages, and lymphocytes. Immunostaining shows AMACR(+), CD10(−), and GATA3(−), consistent with papillary renal cell carcinoma. Periaortic lymph nodes are replaced by fibrous tissue, and the normal lymph node architecture is no longer apparent. No cancer cells are observed. Cholesterin granulation is observed without cancer.

A 35‐year‐old man was referred to our department by a nephrologist for a follow‐up of autosomal dominant polycystic kidney disease. Urinary occult blood was evaluated at presentation to a previous department. The patient required follow‐up because the results suggested autosomal dominant polycystic kidney disease. Subsequently, gross hematuria developed and inflammation persisted. A contrast‐enhanced CT evaluation revealed a neoplastic lesion extending from the left renal parenchyma to the renal pelvis (Fig. 1). Papillary RCC as well as renal pelvic carcinoma with lymph node metastasis were considered as possible diagnoses. A percutaneous needle biopsy was performed, and the results confirmed the diagnosis of RCC; however, a histological diagnosis could not be determined based on the subtype. The clinical diagnosis was cT3aN1M0 with intermediate risk (according to the International Metastatic Renal Cell Carcinoma Database Consortium classification) and a Karnofsky performance score of 90%. Because hilar lymph node metastasis involved the renal artery (maximum diameter, 40 mm), radical hilar treatment was deemed difficult; therefore, combination therapy comprising lenvatinib and pembrolizumab was initiated (lenvatinib 20 mg/day and pembrolizumab 200 mg/3 weeks). Three months after treatment initiation, a CT evaluation revealed that the mass lesion in the left renal parenchyma that extended to the renal pelvis and para‐aortic lymph nodes was smaller. Additionally, gross hematuria was no longer observed. A CT evaluation performed 6 months later showed further reduction of the tumor and lymph nodes (Fig. 2). Adverse events observed with lenvatinib included hand‐foot syndrome (grade 2), hoarseness (grade 1), stomatitis (grade 1), and diarrhea (grade 1). All of these adverse events were acceptable. Adverse events were not observed with pembrolizumab. Lenvatinib 20 mg/day was continued. After completing nine courses of pembrolizumab, resection was possible; therefore, robot‐assisted radical nephrectomy and lymph node dissection were performed. The operative time was 204 min, and the console time was 164 min. Minimal blood loss occurred. The pathological examination results indicated papillary RCC, World Health Organization/International Society of Urological Pathology classification grade 2, no sarcomatoid changes, no lymphovascular invasion, negative resection margins, and stage ypT1aN0. The para‐aortic lymph nodes had firm adhesions, but they were dissected from the arteriovenous system. Additionally, the pathological examination results indicated that the lymph nodes were devoid of viable cells and comprised scar tissue (Fig. 3). Because the patient was young, the possibility of hereditary renal carcinoma was considered. Additionally, because of the presence of papillary renal cell carcinoma, hereditary leiomyomatosis renal cell carcinoma was considered as a differential diagnosis. Although pathological immunostaining for fumarate hydratase and S‐(2‐succino) cysteine was necessary to confirm the diagnosis, it could not be performed for this case. There were no evaluable lesions during the postoperative CT evaluation. Complete resection (both pathologically and clinically), was considered. Recurrence was not observed during the follow‐up CT evaluation at 1 year postoperatively. The patient is currently undergoing observation without postoperative therapy.

## Discussion

Because of the advent of TKI therapy, the treatment of metastatic RCC has evolved significantly. Additionally, because of the advent of ICIs, the results of IO+IO and IO+TKI therapies have improved.^1^, ^2^, ^3^, ^4^, ^5^ However, most clinical trials have mainly focused on ccRCC, and the treatment of nccRCC has been planned according to the protocol for ccRCC. A study that included a phase II trial of IO+TKI therapy for nccRCC reported that KEYNOTE‐B61 demonstrated the benefits and safety of lenvatinib and pembrolizumab combination therapy for nccRCC; furthermore, the objective response rate of the overall cohort was 49%, and the CR rate of patients with papillary histology was 9%.^6^ Combination therapy comprising cabozantinib and nivolumab is also beneficial for nccRCC.^7^ A recent report described complete resection of papillary renal carcinoma after combination therapy comprising cabozantinib and nivolumab for embolization of the inferior vena cava.^8^

Tsimafeyeu *et al*. reported that 23% of patients with papillary RCC expressed fibroblast growth FGFR2 and that the FGFR2‐positive group had significantly shorter progression‐free survival and overall survival after primary therapy compared to those of the FGFR2‐negative group.^9^ Among TKIs, lenvatinib, which inhibits FGFR1‐4 and vascular endothelial growth factor receptor, may be useful as first‐line treatment for metastatic papillary RCC.^10^ Additionally, papillary RCC and chromophobe RCC are nccRCC types that are associated with less immune cell infiltration and require tailored treatment options.^11^ However, Singla *et al*. reported that treatment comprising CN and IO therapy was superior to IO therapy alone for metastatic ccRCC; furthermore, they reported that the pT stage, tumor size, and lymphatic invasion rate were reduced when CN was performed after IO therapy. However, they did not observe the same reductions when IO therapy was administered after CN.^12^ The results of deferred CN after IO+TKI therapy are not yet clear; therefore, additional clinical trials are required. Radical surgery had been deemed impossible for our case because of lymph node metastasis to the renal hilus; however, deferred CN was performed after 6 months of primary therapy comprising lenvatinib and pembrolizumab. The pathology examination of the resected specimen indicated negative margins and a down‐staged primary tumor classification, with only a small amount of RCC remaining in the fibrous tissue. Additionally, lymph node metastasis was replaced by fibrous tissue, the normal lymph node architecture was no longer apparent, and no cancer cells were observed.

## Conclusion

The efficacy of IO+TKI therapy for nccRCC has been mainly described by case reports.^8^ To the best of our knowledge, this is the first report of nccRCC with lymph node metastasis treated with lenvatinib and pembrolizumab combination therapy before CN that resulted in complete resection.

## Author contributions

Takayuki Hosoi: Conceptualization; writing – original draft; writing – review and editing. Shunsuke Yoshioka: Data curation; investigation. Akari Hiraguri: Data curation. Yusuke Kirihana: Data curation. Tomoyuki Koguchi: Data curation. Makoto Tamaki: Supervision. Chiharu Irisawa: Supervision; writing – review and editing.

## Conflict of interest

The authors declare no conflict of interest.

## Approval of the research protocol by an Institutional Reviewer Board

Not applicable.

## Informed consent

Written informed consent for publication was obtained from the patient.

## Registry and the Registration No. of the study/trial

Not applicable.
